# Enhanced target-specific delivery of docetaxel-loaded nanoparticles using engineered T cell receptors†

**DOI:** 10.1039/d1nr04001d

**Published:** 2021-08-20

**Authors:** William J. McDaid, Nikolai Lissin, Ellen Pollheimer, Michelle Greene, Adam Leach, Peter Smyth, Giovanna Bossi, Daniel Longley, David K. Cole, Christopher J. Scott

**Affiliations:** The Patrick G Johnston Centre for Cancer Research, Queen's University Belfast Belfast UK, BT9 7AE c.scott@qub.ac.uk; Cancer Research UK Manchester Institute Alderley Park Congleton Rd Alderley Edge Macclesfield UK SK10 4TG; Immunocore Ltd 101 Park Dr Milton Abingdon United Kingdom OX14 4RY; Institute of Cancer Research 15 Cotswold Rd Sutton London SM2 5NG UK

## Abstract

For effective targeted therapy of cancer with chemotherapy-loaded nanoparticles (NPs), antigens that are selective for cancer cells should be targeted to minimise off-tumour toxicity. Human leukocyte antigens (HLAs) are attractive cancer targets as they can present peptides from tumour-selective proteins on the cell surface, which can be recognised by T cells *via* T cell receptors (TCRs). In this study, docetaxel-loaded polymeric NPs were conjugated to recombinant affinity-enhanced TCRs to target breast cancer cells presenting a tumour-selective peptide-HLA complex. The TCR-conjugated nanoparticles enabled enhanced delivery of docetaxel and induced cell death through tumour-specific peptide-HLA targeting. These *in vitro* data demonstrate the potential of targeting tumour-restricted peptide-HLA epitopes using high affinity TCR-conjugated nanoparticles, representing a novel treatment strategy to deliver therapeutic drugs specifically to cancer cells.

## Abstract 2

High-affinity TCRs offer the potential to target chemotherapeutic drug-loaded nanoparticles specifically to tumour cells expressing tumour-specific peptides minimising off-target effects.

## Graphical abstract

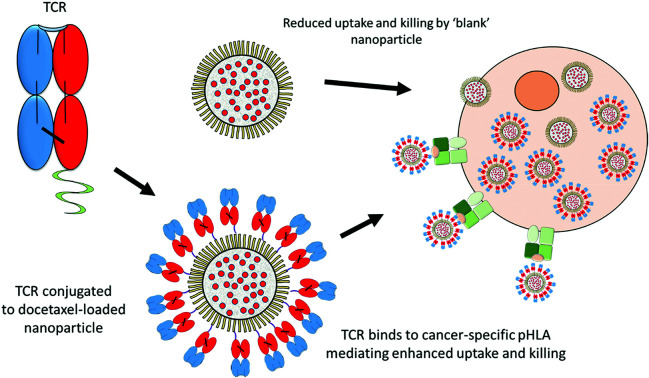

## Introduction

The goal of targeted cancer therapy is to eliminate tumour cells minimising off-target effects in healthy tissues. Typically, cell surface or intracellular proteins found in hyperactive pro-survival pathways are chosen as potential candidates for pharmacological targeting.^1^ One method of targeting these pathways is *via* the human leukocyte antigens (HLAs), as these molecules present protein fragments at the cell surface (normally 8–11 amino acid peptides) that represent the entire cellular proteome.^2,3^ Thus, peptide-HLA (pHLA) complexes presenting unique or dysregulated tumour proteins can be targeted using tumour-specific, or tumour-selective approaches.

The natural ligand of pHLA is the T cell receptor (TCR), the primary antigen receptor expressed on the surface of T cells that governs T cell activation and can lead to killing of target cells.^4^ The ability of the TCR to recognise disease-associated pHLA, including tumour associated epitopes, has led to the development of soluble TCR bispecifics that can redirect T cell activation against specific targets. Although this pathway is very attractive for therapeutic targeting, there are some key limitations: first, tumour associated pHLA can be expressed at very low levels on tumour cells (10s–100s of copies); and second, native TCRs have relatively weak affinities for cognate pHLA (*K*_D_ = μM).^5^ To overcome these issues, tumour-selective TCRs have been affinity-enhanced using directed evolution approaches to generate TCRs that can bind pHLA with picomolar affinities and binding half-lives of several hours.^6^ These affinity-enhanced TCRs have been used to develop ImmTAC (immune mobilised monoclonal TCRs Against Cancer) molecules; bispecific fusion proteins composed of a recombinant affinity-enhanced TCR and an anti-CD3 single chain antibody fragment (scFv). These ImmTAC molecules can specifically target tumour cells expressing very low copy number of pHLAs and, through the anti-CD3 antibody component, redirect polyclonal T cells to tumour cells to elicit an anti-tumour response.^7,8^ Importantly, by redirecting T cells *via* CD3 engagement (which triggers T cell activation *via* the TCR), these reagents are able to maintain the exquisite native levels of sensitivity observed for T cell activation *via* natural TCR-pHLA engagement (T cells can be triggered against >10 antigens).^9^

An alternative application of these affinity-enhanced TCRs could be to enhance the delivery of chemotherapeutic drugs, for example in drug-loaded nanoparticles (NP). This would enable the targeting of cancer-associated pHLA to deliver an amplified payload because of the potential of NPs to transit high amounts of chemotherapy to tumour sites.^10,11^ For example, NPs can be employed as delivery vehicles for anti-cancer agents and through encapsulation, the drug cargo is protected from metabolic degradation and clearance by immune cells through PEGylation. Moreover, it has also been shown that it is possible to actively target NPs through functionalisation of the surface with ligands which bind to proteins usually found overexpressed on the surface of cancer cells. This includes antibodies,^12,13^ oligonucleotides^14,15^ and peptides,^16^ ligated to the NP corona to increase NP targeting and drug delivery *in vivo*. The drawback of this approach is that cell surface targets are often also expressed on normal healthy cells, albeit at lower levels,^17^ potentially resulting in unwanted toxicities. Therefore, unique targeting agents which can recognise and bind exclusively to cancer-associated antigens, such as tumour antigen presenting HLAs, are necessary to improve on conventional targeting strategies which usually depend on the expression cell surface proteins commonly shared between normal and cancerous cell types.

Herein, we report the conjugation of an affinity-enhanced TCR to the surface of docetaxel (DTX)-loaded NPs and assessed the targeting benefit that these TCRs offer to NPs. Following synthesis and characterisation of TCR-conjugated NPs, these reagents were shown to specifically bind to the cognate pHLA complex and improve NP internalisation and DTX efficacy in breast cancer cells *in vitro*.

## Methods

### Construct design, protein expression and purification

To generate high affinity TELa13b1 TCRs for NP conjugation, a cysteine residue was introduced into the TCR β-chain. The modified TCRs, β2m and the HLA-A*02:01 heavy chain were cloned into the pGMT7 vector and expressed in the NEB5α cells *E. coli* strain as described previously.^18^ The TELa13b1 TCR and HLA-A*02:01 in complex with the cognate TELa13b1 TCR hTERT_540–548_ peptide (ILAKFLHWL, A2-ILA from hereon in), or the MAGE-2_277–286_ peptide (ALIETSYVKV, A2-MAGE from hereon in) were refolded from insoluble inclusions bodies and purified using ion exchange and gel filtration as previously described.^19^

### Sodium dodecyl sulfate-polyacrylamide gel electrophoresis (SDS-PAGE)

TCRs were reduced in 0.1 mM dithiothreitol (DTT) and added to Laemmli buffer (Bio-Rad) containing 5% mercaptoethanol (Bio-rad) and were loaded into a Novex 4–20% Tris-glycine SDS-PAGE gel (ThermoFisher Scientific) and were subsequently stained with Coomassie Brilliant Blue.

### Preparation of NPs

NPs were formed using a blend of poly(lactic-*co*-glycolic acid) (PLGA) 502H (Evonik Rohm GmbH) and PEG-PLGA-maleimide (Akina PolySciTech) in a 75% : 25% ratio. 20 mg of the polymer mixture and either 100 μg of docetaxel (DTX) or 50 μg rhodamine-6G were dissolved in 1 mL of dichloromethane (DCM) and injected using a 25G needle into 7 mL of 0.5% (w/v) polyvinyl alcohol/2-morpholinoethanesulfonic acid monohydrate (PVA/MES) buffer at pH 5. This solution was emulsified by pulsar sonication on ice for 90 seconds at an amplitude of 50%, using a Model 120 sonic dismembrator (Fisher Scientific). The resultant NP suspension was stirred overnight at 300 rpm to allow DCM to evaporate. NPs were purified by washing three times in sterile PBS, followed by centrifugation at 20 000*g* for 15 minutes to remove non-entrapped payload before further use.

### Surface conjugation of high affinity TCRs

TELa13b1 TCR was treated overnight with 0.1 mM DTT to reduce disulpfide bonds to expose cysteine residues necessary for site-specific conjugation to NPs. Reduced TCRs were run through gel filtration through a Superdex S75 column to remove DTT. PEG-PLGA-maleimide NPs were resuspended at 2 mg mL^−1^ in PBS containing 100 μg mL^−1^ TCRs. TCR conjugation was carried out overnight on a rotator at room temperature. NPs were then washed in PBS three times to remove unbound TCR by centrifugation at 13 000*g* for 15 minutes at 4 °C. TCR conjugation efficiency was measured using a BCA assay in accordance with the manufacturer's protocol.

### Assessing DTX entrapment by high performance liquid chromatography (HPLC)

DTX-NPs were lysed in acetonitrile : dimethyl sulfoxide solution and were run through a C18 reverse phase column (Phenomenex, 150 mm × 4.6 mm, 5 μM). A standard calibration curve was generated by spiking 5 μg of DTX into a solution of 1 mg mL^−1^ of blank NPs dissolved in ACN–DMSO (1 : 1 volume ratio). The gradient profile of the mobile phase runs from 10% acetonitrile in water to 100% acetonitrile with 0.1% trifluoroacetic acid for 8 minutes at a flow rate of 1.2 mL per minute. DTX was detected at 227 nm and a calibration curve was calculated by measuring the area under integrated chromatographic peaks for DTX standards, from which DTX entrapment was extrapolated.

### Nanoparticle characterisation: size, PDI and zeta potential

NPs were characterised using the NanoBrook Omni Particle Sizer and Zeta Potential Analyzer. NPs were diluted to a concentration of 0.1 mg mL^−1^ in deionized water and size, polydispersity index (PDI) and zeta potential were measured. For visualization, 5 mg mL^−1^ NPs were spotted on aluminium stubs coated with gold and then imaged using a FEI Quanta 250 FEG environmental scanning electron microscope (SEM). Further measurements of size were carried out by NanoSight – NPs were resuspended at 0.1 mg mL^−1^ in deionised water and assessed using Nanoparticle Tracking Analysis (NTA, Malvern NS30). Measurements were conducted in triplicate.

### Surface plasmon resonance (SPR)

NP binding to target pHLA was analysed on a Biacore 3000 instrument (GE Healthcare). Recombinant A2-ILA, or A2-MAGE as a negative control, were immobilised onto a CM5 sensor chip (GE Healthcare) *via* carbodiimide chemistry as previously reported.^20^ Thereafter, 0.1–1 mg polymer per mL NPs diluted in PBS were injected over the A2-ILA-coated chip at a flow rate of 10 μL per minute.

### Cell culture

MDA-MB-231 and MCF-7 cells were purchased from ATCC and were cultured in high glucose (4.5 g L^−1^) Dulbecco's Modified Eagle's Medium (DMEM) supplemented with 50 units per mL penicillin,50 μg mL^−1^ streptomycin and 10% fetal calf serum (FCS). All media and supplements were purchased from Thermo Fisher Scientific.

### Cell surface HLA expression

To measure HLA cell surface expression, cells were stained with either 2 μg FITC-conjugated anti-HLA-A,B,C antibody (Biolegend; 311404) or 2 μg FITC-conjugated IgG2a Isotype control (Biolegend; 400208) for 45 minutes at 4 °C in cell staining buffer (PBS/5% FCS) and analysed by flow cytometry using BD FACS Diva.

### Measurement of NP binding to the cell surface

Cells were either seeded in a black 96-well plate (for measurement of NP binding using a plate reader) or in a 6-well plate (for measurement of NP binding by flow cytometry). Prior to NP treatment, cells were incubated with either ILAKFLHWL or KIFEMLEGV peptides in serum-free media for 2 hours and unbound peptide was washed off with PBS and cells were incubated in fresh media. Cells were then chilled to 4 °C and treated with 25 μg mL^−1^ fluorescent rhodamine-6G loaded NPs for 30 minutes. Cells were then washed three times with ice-cold PBS to remove non-bound NPs. For measuring total fluorescence, cells were lysed in 0.2 M NaOH/0.05% Triton X-100 and fluorescence was measured at 525/555 Ex/Em using a plate reader. Fluorescence was also measured by flow cytometry using a BD FACS Diva instrument.

### Assessing cell viability and apoptosis

Cell viability was measured using the CellTiter-Glo assay (Promega) 48 hours after NP treatment. Viability was calculated as a percentage relative to the untreated control. Apoptotic cell death after 48 or 72 hours was measured by Annexin V and propidium iodide (PI) staining (MDA-MB-231 cells) or by TMRE staining (MCF-7 cells) followed by flow cytometry (BD Accuri C6).

### Assessing colony formation

Cells were initially seeded at 2.5 × 10^5^ cells per well in 6-well plates and incubated with ILAKFLHWL and KIFEMLEGV peptides for 1 hour. After 1 hour, the cells were washed with PBS and exposed to NPs for 30 minutes at 4 °C. Then, the cells were washed again with sterile PBS and re-seeded at 250 cells per well in 6-well plates and were incubated for 13 days to allow colonies to form. The cells were then stained with 0.4% crystal violet for 10 minutes and the number of colonies were counted.

### Cell cycle progression

After a 24-hour NP treatment, dead cells were collected by removing media and pooled with live cells which were detached using 1 mL of PBS/0.1% EDTA. Cells were fixed using ice-cold ethanol. Cells were exposed to RNase A digestion and were stained with PI and analysed by flow cytometry using a BD LSR II instrument.

## Results

### Characterisation of TCR-conjugated nanoparticles for targeted chemotherapy

**Fig. 1:**
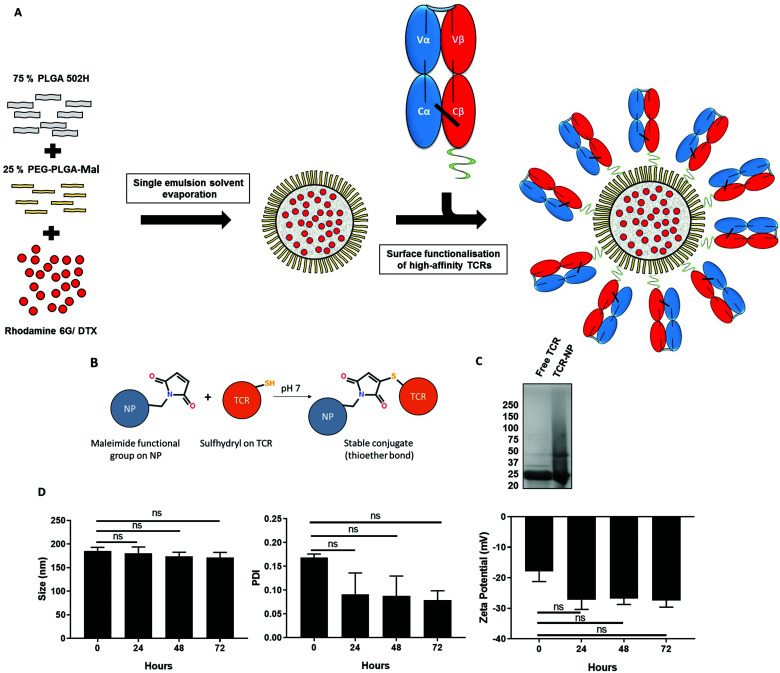
Preparation and characterisation of TELa13b1 TCR-NPs. (A) Schematic overview of the preparation of TELa13b1 TCR-conjugated PLGA NPs using the single emulsion solvent evaporation method. PLGA 502H and PEG-PLGA-maleimide polymers were dissolved together at a ratio of 75% : 25%. Rhodamine 6G or DTX, which were dissolved with the polymers simultaneously, comprised the NP payload. The resultant NPs display PEG chains incorporated into the NP corona which contain maleimide functional groups for the conjugation of the TELa13b1 TCR to the surface. The TELa13b1 TCR was modified with an exposed cysteine residue on the C-terminus of the β-chain for site-specific conjugation to NPs. (B) Maleimide functional groups covalently bind to cysteine sulfhydryl groups on the β-chain of the TELa13b1 TCR forming a thioether linkage resulting in a functionalised NP with TCRs orientated correctly to facilitate binding to pHLA molecules. (C) Covalent attachment of TELa13b1 TCR to PEG-PLGA-maleimide PLGA NPs was confirmed by running 1 mg of TELa13b1 TCR-NPs and an equivalent amount of free TCR on an SDS-PAGE gel under reducing conditions and stained with Coomassie blue. TELa13b1 TCRs were reduced into α- and β-chains (25 kDa). (D) NP stability was assessed by incubating Blank-NPs in FCS-supplemented DMEM at 37 °C and size, polydispersity index (PDI) and zeta potential were measured every 24 hours over a 72-hour period. Mean ± s.e.m. (*n* = 2). Statistical significance determined the using one-way ANOVA and Tukey's *post-hoc* test.

We have previously developed maleimide-functionalised polymeric NPs using a single emulsion-solvent evaporation approach and showed that free thiol ligands may be site-selectively coupled to their surface in a highly efficient manner.^21^ In accordance with this methodology, a blend of PLGA 502H and PEG-PLGA-maleimide polymers was dissolved with a drug or fluorescent marker into an organic solvent, which was subsequently emulsified to produce a loaded NP suspension. We used taxane-based chemotherapeutic docetaxel (DTX) as a model drug or rhodamine 6G (Rho) as a fluorescent marker as its high hydrophobicity results in efficient entrapment. Thereafter, the TELa13b1 TCR (an affinity-enhanced TCR that recognises the HLA-A*02:01 restricted hTERT_540–548_ ILAKFLHWL peptide pHLA complex, (subsequently referred to as A2-ILA), with *K*_D_ = 115 pM (ref. 22)) was conjugated to the surface of the loaded NPs (Fig. 1A). Mild reduction using 0.1 mM DTT reacts with and frees the unpaired cysteine on the β-chain of the TELa13b1 TCR allowing a thioether covalent bond to form with a maleimide functional group on the surface of the nanoparticle (Fig. 1B). Characterisation of newly synthesised NPs by dynamic light scattering (DLS) revealed formulations were within the range of 194.45 ± 4.04 to 214.86 ± 8.1 nm in diameter, with unloaded (blank) and rhodamine-loaded NPs exhibiting smaller diameters and DTX-loaded formulations exhibiting larger diameters. Polydispersity indices (PDIs) were all below 0.12, indicative of monodisperse formulations with a uniform size distribution, while negative zeta potentials were in the range of −15.9 ± 2.16 to −33.72 ± 2.9 mV, suggesting that the formulation was stable and unlikely to aggregate. Scanning electron microscopy (SEM) of blank maleimide NPs confirmed NP sizes of approximately 200 nm (ESI Fig. 1A†). NP tracking analysis of blank-NPs and DTX-NPs also supported DLS analysis showing that both formulations were approximately 200 nm in diameter (ESI Fig. 1B†). DTX encapsulation was measured by HPLC and TELa13b1 TCR were detected on the NP surface and quantified by BCA assay confirming that a TCR-conjugated nanoformulation with a drug cargo had been successfully developed (Table 1). Covalent attachment of TELa13b1 TCR to the surface of NPs was confirmed by the stunted electrophoretic movement of NP-conjugated TCR protein due to the slower migration of NPs through a denaturing SDS-PAGE gel (Fig. 1C). The stability of the NP suspension over time was confirmed by the absence of significant changes to NP size, PDI and zeta potential within a 72-hour period (Fig. 1D).

**Table 1:** NP characterisation. Size, polydispersity index (PDI) and zeta potential were measured in dH_2_0 using the Brookhaven. DTX entrapment was quantified by HPLC and TCR conjugation was measured using a BCA assay. Mean ± s.e.m. (*n* = 3)

NP characteristic	Blank-NP	TCR-Rho-NP	DTX-NP	TCR-DTX-NP
Diameter (nm)	194.45 ± 4.04	193.12 ± 1.27	214.86 ± 8.1	201.56 ± 11.28
PDI	0.076 ± 0.005	0.096 ± 0.019	0.121 ± 0.011	0.115 ± 0.016
Zeta potential (mV)	−15.9 ± 2.16	−20.91 ± 3.45	−33.72 ± 2.9	−21.88 ± 4.19
TCR conjugation (μg mg^−1^)	NA	11.28 ± 2.1	NA	11.28 ± 2.1
DTX loading (μg mg^−1^)	NA	NA	2.22 ± 0.08	
DTX loading efficiency (%)	NA	NA	44.4	

### NP-conjugated TELa13b1 TCRs retain native TCR binding activity

**Fig. 2:**
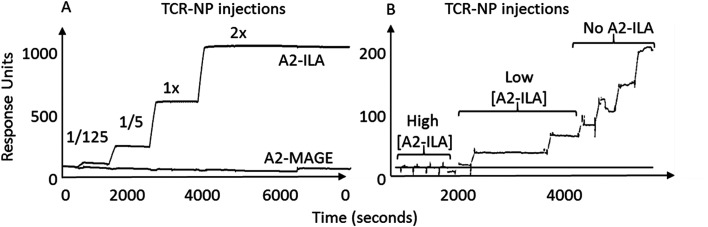
Nanoconjugated TELa13b1 TCR binds specifically to cognate pHLA molecules. (A) Representative sensorgram depicting binding activity of TELa13b1 TCR-Rho-NPs functionalised with 5-fold increasing conjugation efficiencies of TELa13b1 TCRs to HLA-A*02:01-ILAKFLHWL (A2-ILA) and control HLA-A*02:01-ALIETSYVKV (A2-MAGE), assessed by SPR. (B) Representative sensorgram depicting specificity of TELa13b1 TCR-Rho-NPs for A2-ILA. TELa13b1 TCR-Rho-NPs were pre-incubated with non-immobilised A2-ILA and were injected into A2-ILA- and A2-MAGE-immobilised flow cells and binding activity was measured by SPR. Data is representative of two independent experiments.

TELa13b1 TCR-Rho-NPs were subjected to surface plasmon resonance (SPR) analysis to determine their binding capability to immobilised A2-ILA. Biacore, which works on the principle of SPR, measures changes in the refractive index of liquid flown over the surface of a chip.^22^ Biotinylated A2-ILA was immobilised onto the surface of a dextran chip, thus allowing the strength of binding and binding kinetics between TELa13b1 TCR and A2-ILA in real-time to be measured through changes in the refractive index. TELa13b1 TCR-Rho-NPs bound specifically to A2-ILA as indicated by an increase in SPR response compared to the control pHLA, A2-MAGE, in which no response was detected. An even stronger binding response was observed when the amount of TELa13b1 TCR loaded on the NP surface was increased (Fig. 2A). Pre-incubation of TELa13b1 TCR-Rho-NPs with a high concentration of non-immobilised A2-ILA (high [A2-ILA]) completely abrogated NP binding to the immobilised pHLA target, while titrating the amount of non-immobilised A2-ILA (low [A2-ILA]) pre-incubated with TELa13b1 TCR-Rho-NPs reversed this effect in a concentration-dependent manner (Fig. 2B). These data highlight the specificity of this targeted formulation for its cognate pHLA and demonstrate that the binding seen was due to a *bona fide* NP-conjugated TCR-pHLA interaction rather than non-specific adherence of NPs to the dextran chip.

**Fig. 3:**
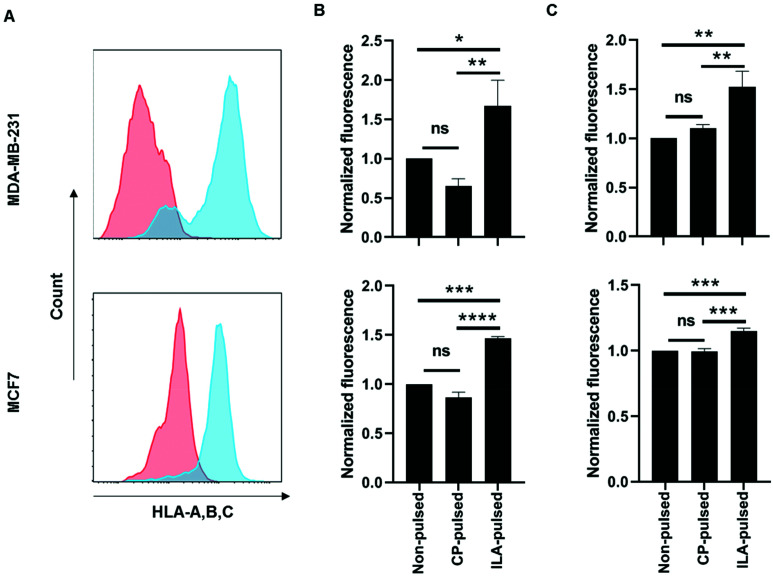
Nanoconjugated TELa13b1 TCR binds to ILAKFLHWL peptide-presenting breast cancer cells *in vitro*. (A) HLA-A,B,C surface expression in MDA-MB-231 and MCF-7 cells was measured by flow cytometry. Red histogram represents cells stained with isotype control antibody; blue histogram represents cells stained with HLA-A,B,C antibody. (B) MDA-MB-231 and MCF-7 cells were either left unpulsed or pulsed with ILAKFLHWL (ILA-pulsed) or control KIFEMLEGV peptides (CP-pulsed) prior to culture with 25 μg mL^−1^ TELa13b1 TCR-Rho-NPs for 30 minutes at 4 °C. Cellular binding of TELa13b1 TCR-Rho-NPs was assessed by measuring rhodamine-6G fluorescence by spectrofluorometry. Data was normalised to mean fluorescence of unpulsed cells and is representative of three independent experiments. Mean ± s.d. (C) MDA-MB-231 and MCF-7 cells were either pulsed with ILAKFLHWL (ILA-pulsed) or control KIFEMLEGV peptides (CP-pulsed) or were not pulsed and exposed to 25 μg mL^−1^ TELa13b1 TCR-Rho-NPs for 30 minutes at 4 °C. Cellular binding of TELa13b1 TCR-Rho-NPs was assessed by flow cytometry through measuring of rhodamine-6G fluorescence. Data was normalised to mean fluorescence of non-pulsed cells and is representative of three independent experiments. Mean ± s.d. Statistical significance for (B) and (C) determined using the one-way ANOVA and Tukey's *post-hoc* test.

Next, the ability of TELa13b1 TCR-NPs to engage A2-ILA on the surface of cancer cells was explored. Two breast cancer cell lines, MDA-MB-231 (triple-negative breast cancer) and MCF-7 (estrogen-positive luminal breast cancer), expressing HLA-A,B,C molecules were selected (Fig. 3A). However, as these cell lines do not naturally process and present ILA peptide within HLA molecules despite expressing hTERT,^23^ they were first pulsed with the ILAKFLHWL peptide to induce pHLA presentation prior to incubation with NPs. Spectrofluorometric analysis of NP binding revealed that ILA-pulsed MDA-MB-231 and MCF-7 cells exposed to rhodamine 6G-encapsulated NPs (TELa13b1 TCR-Rho-NP) demonstrated significantly higher total cellular fluorescence compared to control conditions in which the cells were incubated unpulsed or pulsed with a control peptide (KIFEMLEGV), indicating that TELa13b1 TCR-Rho-NPs bound to the cell surface through A2-ILA engagement (Fig. 3B). This finding was further confirmed by flow cytometry (Fig. 3C), highlighting that NP-conjugated TCRs are capable of engaging cognate pHLAs *in vitro* and that the effect is dependent on the display of the target peptide species in the HLA complex.

### TELa13b1 TCR-DTX-NPs engage A2-ILA to promote breast cancer cell apoptosis

**Fig. 4:**
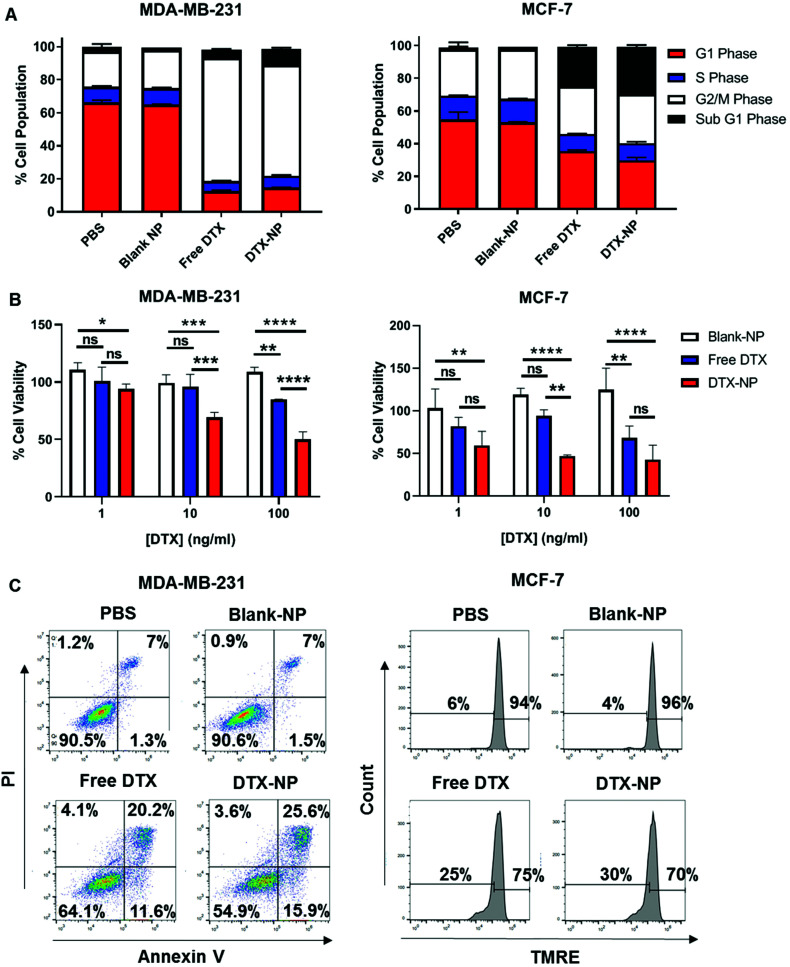
Nanoencapsulation of DTX within PLGA NPs improves efficacy. (A) Flow cytometric analysis of cell cycle progression in MDA-MB-231 and MCF-7 cells 24 hours after treatment with DTX-NPs at a final concentration of 10 ng DTX per mL and equivalent amounts of either free DTX or Blank-NPs. Data presented as a quantification of MDA-MB-231 and MCF-7 cells in each phase of the cell cycle. Data representative of three independent experiments (B) Viability of MDA-MB-231 and MCF-7 cells 48 hours following treatment with increasing concentrations of DTX-NPs and equivalent amounts of free DTX and blank-NPs measured by CellTiter-Glo. Data expressed as a percentage of PBS control and is representative of three independent experiments. Mean ± s.d. Statistical significance determined using the two-way ANOVA and Tukey's *post-hoc* test. (C) Flow cytometric analysis of cell death in MDA-MB-231 and MCF-7 cells 48 hours following treatment with DTX-NPs at a final concentration of 10 ng DTX per mL and equivalent amounts of either free DTX or Blank-NPs. For measurement of cell death, MDA-MB-231 cells were stained with Annexin V and PI and MCF-7 cells were stained with TMRE. Data representative of three independent experiments.

Following validation of the targeting functionality of TELa13b1 TCR-NPs, DTX was encapsulated into non-targeted NPs to assess efficacy *in vitro*. Cell cycle profiling revealed that both free DTX and nanoencapsulated DTX had similar impacts on cell cycle distribution at 24 hours post-exposure to the drug. In MDA-MB-231 cells, free and encapsulated DTX caused cells to arrest in the G2/M phase of the cell cycle to an equal extent. In MCF-7 cells, free and encapsulated DTX increased the percentage of MCF-7 cells in sub-G1 phase to an equal extent (Fig. 4A). Interestingly at longer time points, nanoencapsulated DTX dose-dependently reduced cell viability to a greater extent than free DTX in both cancer cell lines (Fig. 4B), which we attribute to a greater percentage of apoptosis in cells exposed to DTX-NPs (41.5% for MDA-MB-231 cells and 30% for MCF-7 cells) compared to free DTX (31.8% for MDA-MB-21 cells and 25% for MCF-7 cells) (Fig. 4C). Collectively, these data suggest that encapsulation of DTX within polymeric NPs does not compromise drug activity and increases its efficacy.

**Fig. 5:**
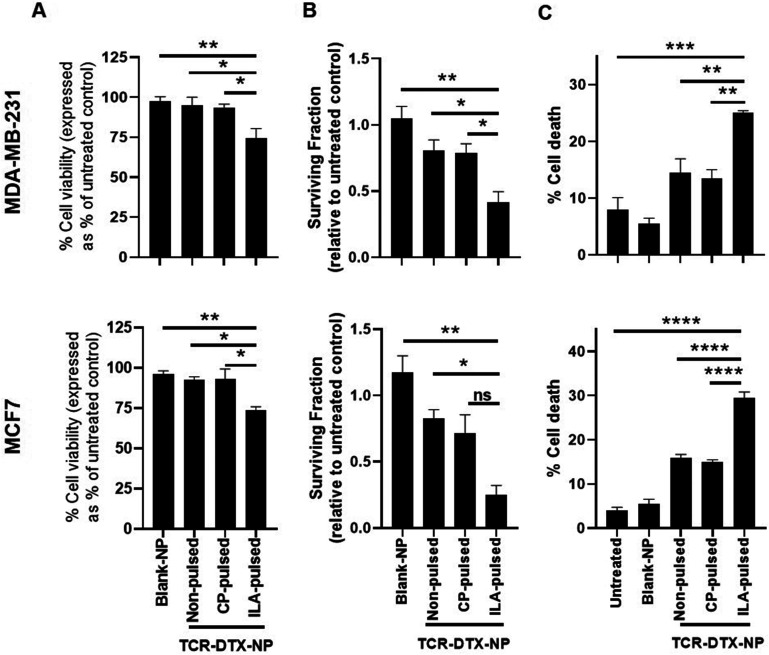
TELa13b1 TCR-DTX-NPs induce cytotoxic effects mediated through TELa13b1 TCR-pHLA engagement. (A) Viability of peptide-pulsed MDA-MB-231 and MCF-7 cells at 48 hours following a 1 hour treatment of 25 μg mL^−1^ TELa13b1 TCR-DTX-NPs containing 55.5 ng mL^−1^ DTX and equivalent amounts blank-NPs at 4 °C. Viability was measured by CellTiter-Glo. Data expressed as a percentage of PBS control. Mean ± s.e.m. (*n* = 3). (B) MDA-MB-231 and MCF-7 colony formation at 10 days following a 1 hour treatment of 25 μg mL^−1^ TELa13b1 TCR-DTX-NPs containing 55.5 ng mL^−1^ DTX and equivalent amounts blank-NPs at 4 °C. Data expressed relative to PBS control and is representative of three independent experiments. Mean ± s.e.m. (*n* = 3). (C) Flow cytometric analysis of cell death in peptide-pulsed MDA-MB-231 and MCF-7 cells at 72 hours following a 1 hour treatment of 25 μg mL^−1^ TELa13b1 TCR-DTX-NPs and equivalent amounts of Blank-NPs at 4 °C. For measurement of cell death, MDA-MB-231 cells were stained with Annexin V and PI and MCF-7 cells were stained with TMRE. Mean ± s.e.m. Statistical significance for (A), (B) and (C) determined the using one-way ANOVA and Tukey's *post-hoc* test. ILA-pulsed (pulsed with ILAKFLHWL peptide), CP-pulsed (pulsed with control KIFEMLEGV peptide).

Next, the TELa13b1 TCR was conjugated to the surface of DTX-NPs and the benefit of pHLA targeting on NP-induced cytotoxicity was investigated. ILAKFLHWL-pulsed, control peptide KIFEMLEGV-pulsed and unpulsed breast cancer cells were exposed to TELa13b1 TCR-DTX-NPs for 1 hour and non-bound NPs were removed by washing the cells with PBS followed by media replacement. After a 48 hours incubation, a reduction in the viability of ILAKFLHWL-pulsed cells was seen with minimal impact on unpulsed cells or cells pulsed with a control peptide (KIFEMLEGV), suggesting that the targeting of A2-ILA by this formulation facilitated greater uptake of nanoencapsulated DTX resulting in increased cell death (Fig. 5A). Similarly, the long-term impact of this formulation revealed a decrease in the number of viable cell colonies in ILAKFLHWL-pulsed cells compared to control cells, further indicating that pHLA targeting increases NP uptake and DTX-induced cytotoxicity (Fig. 5B). Finally, flow cytometric analysis demonstrated that TELa13b1 TCR-DTX-NPs induced significantly more apoptosis in cells pre-pulsed with ILAKFLHWL peptide compared to control cells (Fig. 5C). Taken together, these data demonstrate that conjugation of TELa13b1 TCRs to DTX-NPs increases the uptake of this formulation, thereby increasing the intracellular concentration of DTX, promoting a higher apoptotic response. Importantly, as both of these cell lines represent different subtypes of breast cancer, these data also suggest that this TCR-conjugated nanotherapeutic has broad applicability and can be used in wider groups of patients.

## Discussion

In this study, the interaction between an affinity enhanced TCR and its cognate pHLA was exploited to enhance the targeted delivery of a DTX-loaded NP formulation to cancer cells *in vitro*. Polymeric NPs were synthesised and conjugated to the TELa13b1 TCR which recognises and binds to A2-ILA. The therapeutic efficacy of this targeted formulation was validated through assessment of DTX delivery and the resulting tumour cell cytotoxicity, demonstrating, for the first time, the potential of affinity-enhanced TCRs as NP targeting conjugates.

The TELa13b1 TCR was conjugated to polymeric NPs using maleimide chemistry. Maleimide functional groups located on PEG chains on the NP corona covalently attached the TELa13b1 TCR to the NPs in a site-specific manner through the exposed cysteine residue engineered onto the TCR β-chain. This site-specific method allowed the antigen binding site of TCR to be accessible for pHLA interactions and overcomes the disadvantages observed with conventional methods such as carbodiimide chemistry. Similar methods of site-specific conjugation strategies, such as strained-promoted alkyne azide,^24^ alkyne-nitrone click chemistry^25^ and UV photo-crosslinking,^26^ have previously been reported to improve NP targeting *in vitro* and *in vivo*, underlining the relevance of employing the strategy in this study. Blank-NPs were shown to be stable at physiological conditions over 72 hours. However, for eventual clinical application more investigation of long term preservation (*e.g.* −80 °C storage) are necessary.

We demonstrated that TELa13b1 TCR-conjugated NPs bound specifically to A2-ILA and TCR loading was directly proportional to the strength of binding. This is supported by a study in which affinity enhanced MAGE A1 TCR-like single chain antibodies were conjugated to the surface of PEGylated immunoliposomes through maleimide chemistry.^27^ Similarly, these liposomes bound specifically to the target antigen highlighting the potential of targeting tumour-associated pHLAs for NP delivery. In this study by Saeed *et al.*, the difference between binding of targeted immunoliposomes and non-targeted immunoliposomes was very dramatic. Despite our data showing a significant increase in binding of TCR-NPs to A2-ILA compared to controls *in vitro*, further studies using different concentrations of NP would allow us to understand in more depth the cell-surface antigen saturation point and the effect on non-specific binding. Interestingly, no dissociation of NP-conjugated TELa13b1 TCR from A2-ILA was detected, which contrasts with previous studies of high affinity TCRs, which typically dissociate slowly over time.^6,23^ This may be attributed to the correct orientation of the NP-conjugated TELa13b1 TCR allowing an avidity effect to arise due to multiple TCR-A2-ILA interactions. It is plausible that the strong binding interaction seen by SPR and the enhanced binding observed *in vitro* may be due to pHLA crosslinking by the NP-conjugated TELa13b1 TCR. This has been reported previously in studies of antibody-conjugated NPs where it was suggested that NP-conjugated antibodies promoted receptor clustering as evidenced by a potentiation of signalling through intracellular pathways downstream of the receptor.^28,29^

DTX is commonly used to treat triple negative breast cancer but is often accompanied by unwanted toxicities such as stomatitis, neutropenia and hypersensitivity reactions.^30^ Thus, nanoencapsulation of this taxane has been studied using different formulations including gold,^31^ chitosan,^32^ polycaprolactone polyesters^33^ and PLGA.^34^ Here, the potency of DTX was enhanced after encapsulation. This finding has been also been reported in other studies,^35,36^ collectively suggesting that NP-mediated delivery of this drug improves its therapeutic index.

By covalently attaching a TCR to DTX-NPs, a greater percentage of breast cancer cells underwent apoptotic cell death than TCR-DTX-NPs targeting unpulsed cells or cells pulsed with the control peptide. This suggests that this specific TCR-pHLA interaction further increased NP internalisation promoting higher DTX-induced cytotoxicity. However, further analysis is required to determine the mechanism and time course for NP uptake and the point of release of the DTX payload after binding to the pHLA complex on the cell surface. TCR-like antibodies have also been conjugated to drug molecules to form antibody–drug conjugates (ADCs), through which a superior apoptotic phenotype was seen after binding of the ADC to cognate antigen-presenting pHLAs.^37^ However, ADCs possess drawbacks such as potential systemic toxicity due to the premature release of the drug conjugate and low drug-to-antibody ratios (DARs). Thus, employing affinity enhanced TCRs as targeting agents for drug-loaded NPs may be a more translatable strategy due to higher DAR ratios and higher drug capacities.^38^ Additionally, TCR-like antibodies have been shown to be less specific than TCRs, raising concerns over off-target toxicity when employing these reagents.^39^

Although we have clearly demonstrated that this TCR-conjugated nanotherapeutic has clinical potential using *in vitro* models, our findings do have some limitations and further study is required. This includes *in vivo* characterisation using *in vivo* models of cancer. Since this formulation is PEGylated, we intend to administer intravenously to show that the protective advantage of incorporating PEG chains on the NP corona which is well known to minimise premature reticuloendothelial system clearance.^40^ Additionally, studies into the efficacy of TCR-NPs in heterogeneous tumour models would further support the applicability of this technology in the clinic. It has been found that upon release of the drug component of ADCs in a tumour cell, bystander cytotoxicity within other tumour cells can occur which can therefore circumvent the issue that some cells either express low or no antibody target.^41^ We suspect that this may prove to be the case for TCR-NPs in the setting that different populations of cancer cells within the tumour express varying levels of HLA. Similarly, docetaxel-induced cell death has been shown to induce bystander effects in tumours^42^ further mitigating against the issue of tumour cell heterogeneity.

## Conclusion

The results shown in this study demonstrate that affinity-enhanced TCRs can be used to improve the specific delivery of NPs dependent on the presentation of cognate pHLA on target tumour cells. This approach has the potential to deliver an amplified payload to antigen-positive tumour cells through encapsulation of chemotherapeutics. These findings provide new possibilities for targeted delivery of tumour therapies using functionalised NPs.

## Author contributions

W. McDaid – experimental design, execution of experiments, manuscript writing and editing. N. Lissin – experimental design and execution of experiments. E. Pollheimer – execution of experiments. M. Greene – experimental design, manuscript writing and editing. A. Leach – execution of experiments. P. Smyth – execution of experiments. G. Bossi – experimental design. D. Longley – experimental design, manuscript editing, acquisition of funding. D. Cole – experimental design, manuscript writing and editing. C. J. Scott – experimental design, manuscript writing and editing and acquisition of funding.

## Conflicts of interest

There are no conflicts to declare.

## Supplementary Material

NR-013-D1NR04001D-s001
