# Supplementary material for: Enhanced target-specific delivery of docetaxel-loaded nanoparticles using engineered T cell receptors
Source: Nanoscale. 2021 Aug 20;13(35):15010–20. doi: 10.1039/d1nr04001d (PMC8447836; doi:10.1039/d1nr04001d)
Supplement: NR-013-D1NR04001D-s001 [file NR-013-D1NR04001D-s001.pdf]

Electronic Supplementary Material (ESI) for Nanoscale.
This journal is © The Royal Society of Chemistry 2021

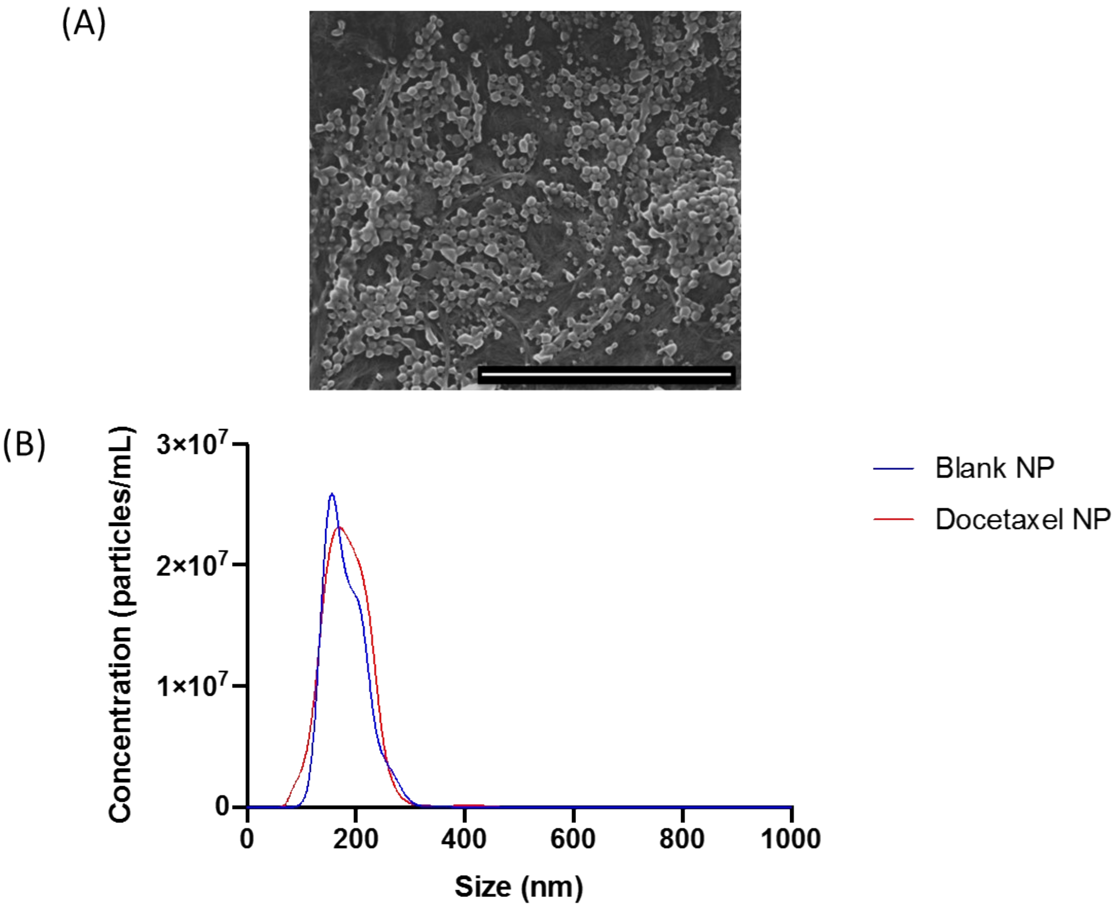

Supplementary Figure 1 – Characterisation of blank-NPs and DTX-NPs. (A) Scanning electron micrograph of blank PEGylated maleimide nanoparticles. Scale bar = 5 µM. (B) Characterisation of size of blank-NPs and DTX-NPs by Nanosight
